# Changes in the salivary electrolytic dynamic after sucrose exposure in children with Early Childhood Caries

**DOI:** 10.1038/s41598-020-61128-6

**Published:** 2020-03-05

**Authors:** Emerson Tavares de Sousa, Aline Tavares Lima-Holanda, Marinês Nobre-dos-Santos

**Affiliations:** Department of Health Sciences and Pediatric Dentistry, Piracicaba Dental School, University of Campinas-UNICAMP, Av. Limeira 901, Piracicaba, SP CEP 13414-903 Brazil

**Keywords:** Diagnostic markers, Risk factors

## Abstract

This study sought to explore if the effect of 20% sucrose rinse (SR) on the salivary electrolytic concentration of calcium (Ca^2+^), phosphate (Pi) and fluoride (F^−^) in children with Early Childhood Caries (ECC) is different from healthy children. Here, fifty-eight  preschoolers aged 3 to 5 years were divided into 2 groups: caries-free (CF) and with ECC. Changes in saliva flow rate, pH and buffering capacity (BC), as well as in concentrations of Ca^2+^, Pi, and F^−^, and the degree of saturation in relation to hydroxyapatite (DSS HAp) and fluorapatite (DSS FAp) were evaluated. The pre-rinse [Ca^2+^] was higher in the ECC group in the CF group. A significant increase in [Ca^2+^] was demonstrated after SR in the CF group (p = 0.05). The [Pi] was reduced by 18% after SR in the ECC group (p = 0.007). The [F^-^] reduced in both groups after SR (p < 0.000). There was a moderate positive correlation between [Ca^2+^] and the DSS HAp and DSS FAp. Multivariate analysis showed that children with a higher [Ca^2+^] in pre-rinse saliva are more likely to have ECC. In conclusion, the effect of a 20% sucrose rinse on the electrolytic concentration of Ca^2+^, Pi and F^-^ was different when children with ECC were compared with CF children.

## Introduction

Early Childhood Caries (ECC) is understood as a chronic, complex and dynamic process of tooth demineralization that can damage irreversibly the tooth crown of children under 6 years old^1,2^. This disease will occur when poor oral hygiene is associated with frequent ingestion of fermentable carbohydrate (mainly sucrose) promoting a shift on the ecology and on the microbial metabolic framework of the biofilm and enhancing the bacteria pathogenicity. Under this perspective, saccharolytic bacteria of the oral cavity initiate an efficient acid production that disrupts the natural equilibrium between the events of demineralization and remineralization of the crystalline mineral structure of the tooth that culminates in the predominance of demineralization events and in the formation of a subsurface caries lesion^2,3^.

The dental hard tissues are composed of inorganic material (96 wt.% for enamel and 70 wt.% for dentin) organized in a crystal structure of hydroxyapatite (Ca_10_(PO_4_)_6_(OH)_2_) and fluorapatite (Ca_10_(PO_4_)_6_F_2_)^4^. In a healthy situation, the inorganic composition of saliva promotes an environment supersaturation of minerals (calcium, phosphate, and fluoride) around and inside the biofilm structure^5^, cooperating to maintain the enamel integrity and to mitigate the damage caused by saccharolytic bacterias^6,7^. From a pragmatic point of view, the speed and direction of the cariogenic partway (Demineralization ↔ Remineralization) are closely associated with the saliva’s ability to maintain the mineral structure of the tooth after a cariogenic challenge with fermentable carbohydrate. Thus, saliva is considered the most important host-related factor that could shift the direction of dental caries activity to the arrested pattern, as well as to promote a significant impact on the maintenance of children’s health^8,9^.

From a biochemical perspective, it has been recognized that changes occurring in the physicochemical properties of saliva, such as on pH and on buffering capacity are expected in the saliva of children with ECC^6,7^. The effect of pH and buffering capacity of saliva promote modification of the bioavailability of calcium, phosphate, and fluoride electrolytes, mainly because fluctuations on the pH change the ionic activity product (IAP) of saliva ions^7,10^. Clinical studies that investigated the electrolytic concentration of calcium, phosphate, and fluoride in the saliva of children have provided a contrasting overview regarding differences between caries-active and caries-free individuals^11–14^. Thus, at least to our knowledge, there is limited evidence to conclude that electrolytic bioavailability of these electrolytes in saliva plays a key role in dental caries disease^15^. In addition, taking the clinical situation as a whole, the ability of saliva to repair enamel after a cariogenic challenge remains unclear, especially because the environmental and physiological factors beyond the ECC can act by modulating the electrolytic behavior of saliva in a high-risk situation for dental caries development. Thus, the available scientific literature did not provide a clear overview of what really happens with the ionic concentration of calcium, phosphate, and fluoride when the pH drops after intake of sweetened food by children with and without ECC.

Given this background, the main objective of this research was to answer the PICO question: Is there any change in the electrolytic concentration of calcium, phosphate, and fluoride after a cariogenic challenger with a 20% sucrose rinse when children with ECC are compared with caries-free children?

## Results

During the sample collection, two volunteers from the CF group dropped out of the study (N = 58: ECC = 30 and CF = 28). The median number of decayed, missing and filled surfaces (dmfs) of the ECC group was 3.00 (Interquartile Range: 11.0), and dmfs plus white spot lesions were 8.00 (Interquartile Range: 12.0). There was no difference between age and gender in the sample (p > 0.05) and the ratio of visible biofilm did not demonstrate any significant difference when CF children and children with ECC were compared. The buffering capacity of saliva is higher in caries-free individuals than in individuals with ECC. (Table 1).

**Table 1:** Sample characteristics according to the ECC experience.

	ECC	CF	p
Age (years) – Mean (SD)	4.67 (0.48)	4.57 (0.57)	0.494^Ψ^
Gender Ratio (M:F)	1.5:1.0	1.33:1.0	0.825^ʊ^
Visible Biofilm Ratio (A:P)	1.0:1.5	1.54:1.0	0.115^ʊ^
Buffering Capacity of Saliva – Median (IQR)	1.32 (0.47)	1.57 (0.42)	0.001^β^

The p-values evidence comparisons between ECC and CF groups (^Ψ^Independent t-test; ^ʊ^Chi-square test; ^β^Mann-Whitnet test).

Table 2 demonstrates that the effect of sucrose rinse influenced SFR, pH, Ca^2+^, Pi and F^−^ concentration, and DSS HAp variables. The Post-hoc LSD test evidence that in the pre-rinse moment, calcium concentration was significantly higher in the saliva of children with ECC than in the saliva of caries-free children. On the other hand, after the sucrose rinse, no difference between groups was noted for SFR, pH, Ca^2+^, Pi and F^−^ concentration, DSS HAp, and DSS FAp. Considering the comparisons between pre- and post-rinse moments, it was observed that the pH and F^−^ concentrations in saliva decreased significantly in both groups after rinse with 20% sucrose (p < 0.01). In the ECC group, a significant increase in SFR was observed, followed by a decrease in the Pi concentration in saliva after the cariogenic challenge. On the other hand, the salivary levels of calcium decreased 14% after the sucrose rinse only in the CF group.

**Table 2:** Main and interaction effect of sucrose rinse and the disease on the flow rate (SFR), pH, Ca^2+^, Pi and F^−^ concentration (µg/mL), and DSS HAp and DSS FAp in saliva: A one-way repeated measures ANOVA.

Repeated Measures (Sucrose Rinse)	Disease		Main Effect				Interaction Effect		Box’ M: p-value
	ECC	CF	Sucrose Rinse		Disease		Sucrose Rinse x Disease		
			p (Power)	ηp²	p (Power)	ηp²	p (Power)	ηp²	
SFR (Pre-rinse)	0.81 (0.49)aA	0.84 (0.44)aA	0.00 (0.95)	0.18	0.64 (0.08)	0.00	0.17 (0.28)	0.03	0.12
SFR (Post-rinse)	1.06 (0.35)aB	0.88 (0.61)aA							
pH (Pre-rinse)	7.60 (0.56)aA	7.67 (0.52)aA	0.00 (0.99)	0.28	0.27 (0.20)	0.02	0.99 (0.05)	0.00	0.90
pH (Post-rinse)	7.32 (0.70)aB	7.45 (0.63)aB							
Ca^2+^ (Pre-rinse)	14.92 (9.04)aA	11.31 (14.48)bA	0.00 (0.77)	0.11	0.08 (0.41)	0.05	0.14 (0.30)	0.04	0.90
Ca^2+^ (Post-rinse)	14.23 (14.48)aA	13.52 (7.63)aB							
Pi (Pre-rinse)	123.81 (50.61)aA	109.23 (33.83)aA	0.03 (0.87)	0.14	0.50 (0.10)	0.00	0.39 (0.13)	0.01	0.23
Pi (Post-rinse)	101.2 (32.45)aB	108.54 (29.94)aA							
F^−^ (Pre-rinse)	0.023 (0.01)aA	0.019 (0.01)aA	0.00 (1.00)	0.53	0.19 (0.26)	0.03	0.39 (0.13)	0.01	0.24
F^−^ (Post-rinse)	0.013 (0.00)aB	0.013 (0.00)aB							
DSS HAp (Pre-rinse)	14.96 (6.91)aA	12.93 (7.64)aA	0.04 (0.52)	0.07	0.52 (0.10)	0.00	0.31 (0.17)	0.09	0.73
DSS HAp (Post-rinse)	12.28 (12.34)aA	11.53 (12.91)aA							
DSS FAp (Pre-rinse)	22.34 (11.26)aA	18.98 (8.39)aA	0.06 (0.46)	0.06	0.91 (0.05)	0.00	0.42 (0.13)	0.01	0.67
DSS FAp (Post-rinse)	18.21 (16.08)aA	16.94 (15.37)aA							

Statistical analyses were performed with a sample of 58 volunteers. ηp²: Partial eta squared.Data were expressed in median (interquartile range). Data were transformed using a logarithmic expression with base 10. Different lower letters represent statistically significant differences between columns (ECC x CF children). Different capital letters represent statistically significant differences in each column (Pre- x Post-rinse). The posthoc LSD (Least Significant Difference) test plus Bonferroni adjustment (p-value has to be smaller than 0.05/4 = 0.0125 to be significant) was used to pairwise comparisons.

A positive correlation between calcium concentration in saliva and the degree of saliva saturation with respect to HAp and FAp were found before and after the cariogenic challenge in children with ECC and in caries-free children (p < 0.001). In addition, a positive correlation between the salivary pH and the degree of saliva saturation before and after sucrose rinse in preschoolers with ECC as well as in caries-free preschoolers (p < 0.001) – Table 3. Data from this table also show that there was no relationship between SFR, BC, Pi, and F^−^ and the degree of saliva saturation before or after sucrose rinse.

**Table 3:** Relationship between physicochemical properties of saliva and the Degree of Saliva Saturation (DSS) with respect to hydroxyapatite (HAp) and fluorapatite (FAp) in the saliva of children with ECC.

	ECC				CF			
	Pre-rinse		Post-rinse		Pre-rinse		Post-rinse	
	DSS HAp	DSS FAp	DSS HAp	DSS FAp	DSS HAp	DSS FAp	DSS HAp	DSS FAp
SFR	—	—	—	—	—	—	—	—
BC	—	—	—	—	—	—	—	—
pH	0.78**	0.71**	0.83**	0.75**	0.77**	0.70**	0.85**	0.79**
Ca^2+^	0.44*	0.53**	0.49**	0.57**	0.49**	0.59**	0.46**	0.55**
Pi	—	—	—	—	—	—	—	—
F^−^	—	—	—	—	—	—	—	—

**The double asterisks represent p-value of Pearson correlation test under 0.001.

Model 1 of the multiple linear regression analysis demonstrates that a higher salivary buffering capacity decreases by 98% the chance of ECC occurrence. In addition, pH and calcium levels can be explanatory variables of salivary behavior in the pre-rinse situation. In Model 2, only the buffering capacity of saliva shows the potential to be an explanatory variable after adjustment for confounders (Table 4).

**Table 4:** Models of multiple logistic regression of ECC before and after a cariogenic challenge with 20% sucrose.

	Model 1 Pre-rinse			Model 2 Post-rinse		
	*Odds Ratio*	CI	p	*Odds Ratio*	CI	p
Age	4.37	(0.79–24.15)	0.09	2.60	(0.63–10.80)	0.19
Gender (Male/Female)	2.90	(0.63–13.29)	0.17	1.28	(0.34–4.29)	0.72
VB (Presence/Absence)	0.317	(0.06–1.56)	0.16	0.51	(0.12–2.10)	0.35
BC	0.021	(0.001–0.31)	0.005	0.05	(0.007–0.40)	0.005
SFR	1.96	(0.22–17.23)	0.55	1.35	(0.33–5.46)	0.68
SpH	0.04	(0.003–0.55)	0.02	0.65	(0.13–3.33)	0.61
Ca^2+^	1.15	(1.00–1.32)	0.048	1.02	(0.95–1.08)	0.62
Pi	0.99	(0.96–1.02)	0.48	1.00	(0.97–1.03)	0.80
F^−^	1.29	(0.83–2.03)	0.25	1.65	(0.19–14.2)	0.65
Constant	0.000		0.09	4.49		0.86

N = 58. Main outcome: ECC; The interpretation of the categorical variables (Gender and VB) should be done using the last category as the reference variable. VB (Visible Biofilm), BC (Buffering Capacity of Saliva), SFR (Salivary Flow Rate), pH (Salivary pH); The overall statistics of Model 1 is 20.48, p = 0.01 (p-value of Hosmer-Lemeshow test = 0.22); the overall statistics of Model 2 is 14.37, p = 0.11 (p-value of Hosmer-Lemeshow test = 0.49).

## Discussion

Evidence from *in vivo* studies demonstrated that several constituents and properties of saliva could affect the caries process^15,16^. Among constituents and properties of saliva, electrolytes (mainly fluoride, calcium, and phosphate), salivary flow rate, buffering capacity and pH are considered of particular importance in the de- and remineralization dynamic. Given this context, investigation of these factors may enhance the understanding regarding the behavior of saliva in individuals who are exposed to frequent cariogenic challenges and have poor oral health. Consequently, the specific ability of saliva to promote a reparative medium for enamel demineralization undergoes remineralization after a cariogenic challenge.

A new and interesting methodological detail of this study was the use of a real-life condition of sugar exposure and its effect on electrolytes. This model includes the use of stimulated saliva promoted by a masticatory stimulus (pre-rinse) and masticatory plus gustatory stimulus (post-rinse). In addition, the use of a specific group of children with a narrow age range, who were subjected to a similar diet (sharing 5 daily meals in the morning and in the afternoon), and who had the same access to fluoridated water (0.71 mg of F/mL) and fluoridated dentifrice.

In the ECC group, a significant decrease was seen in the phosphate and fluoride concentrations in saliva after the cariogenic challenge. In contrast, in the CF group, the salivary levels of calcium increased 14% and the fluoride concentration decreased 31% after the sucrose rinse. Moreover, before the cariogenic challenge, the calcium concentration was markedly lower in the CF group than in the ECC group. Clinical studies that investigated the ionic concentration of calcium, phosphate, and fluoride in the saliva of children have provided a contrasting overview regarding differences between caries-active and caries-free individuals. Most of these studies found that there was a higher calcium, phosphate and fluoride concentration in saliva of CF children^11–13^. However, it should be noted that in these studies the authors used unstimulated saliva as a predictor and did not consider the immediate effect of sucrose ingestion. Moreover, considering the behavioral aspects of childhood and the influence of environmental factors in the development of ECC, it is possible that the findings of this study have potential validity because they reflect a real daily situation since individuals of this study were exposed to the same fluoride sources. More importantly, the low age range of children can markedly reduce the differences in saliva composition found during the maturation of salivary glands.

Regarding the higher calcium concentration in saliva of children with ECC when compared with CF children pre-rinse, there are two possible explanations for this finding. Firstly, individuals with ECC have an increase in H^+^ levels in saliva modulated by the biofilm accumulation and frequent ingestion of fermentable sugars, which can promote a greater mobilization of calcium caused by the increased hydroxyapatite dissolution in the oral cavity. In the same way, a similar electrolytic behavior was observed in pooled plaque samples in which the pH drop after sucrose rinse was followed by an increase in lactate and calcium concentrations^17–19^. It was also demonstrated that the exopolysaccharide matrix in the biofilm can regulate calcium flow in cariogenic biofilms^20^. Secondly, it is likely that salivary proteins interact in the oral environment and form micelle-like structures, which have asymmetrical charge distribution (amphiphilic molecules) with great potential to attract calcium phosphates. Thus, these micelles act maintaining calcium supersaturation in saliva. During a pH fall, it is possible that the charge distribution of these micelles modifies their colloidal stability^21^. From this perspective, the effects of pH drop on these protein complexes and their direct effect on the electrolytic shift on the oral cavity of individuals with ECC and caries-free could be an interesting subject for future research.

The calcium levels in the saliva of children with ECC remained high after sucrose rinse. These results may be a consequence of the metabolic capacity of cariogenic biofilm in maintaining sites of low pH in biofilm and of its sugar catabolic activity during periods of nutrient limitation in the oral cavity using intracellular and extracellular polysaccharides as a sugar reservoir. Consequently, the metabolism of intracellular and extracellular polysaccharides can promote a greater susceptibility to dental caries by prolonging the exposure of tooth surfaces to organic acids. In other words, there is an upregulation mechanism for dental demineralization and consequently a constant generation of acidic pH niches^22–24^. With regard to the CF group, and according to the latter-mentioned hypothesis, the increase in the salivary calcium concentration after sucrose rinse can be explained by the low basal metabolism of the biofilm in CF individuals, which is forced to increase the sucrose metabolism and ultimately shift the ionic imbalance at the interface tooth-biofilm-saliva.

Considering that the dynamic process of caries development starts with the intake of fermentable carbohydrates, cariogenic challenge promotes an important variation on the behavior of salivary functions and on the kinetics of its electrolytes, modifying the ability of the oral environment to reverse the deleterious effect of pH drops^6,7^. Taking this into account, the results of this investigation showed that children with ECC exhibited a deeper pH fall 5 minutes after the sucrose exposure than caries-free children (ECC: ΔpH_5min_ = 0.28 (0.14) and CF: ΔpH_5min_ = 0.22 (0.11); p = 0.05). In addition, the buffering capacity of saliva was higher in individuals without ECC (p = 0.001). As a consensus, the pH and buffering capacity properties of saliva have important relevance in dental caries dynamic, which can be supported by *in vitro* and *in vivo* studies^16,25–27^. Hence, it can be speculated that the risk behavior concerning ECC could enhance caries progression by a linked mechanism of intraoral pH control. The pH control in the oral cavity is ruled by ionic and protein systems that interact in a complex environment to allow a quick return to a neutral pH after a cariogenic challenge^18^. In line with this thought, the continuous exposure of the oral environment to fermented carbohydrates could weaken the physiological ability of saliva resisting to pH drops.

From a clinical perspective, the tendency of the ECC group to have an increase in hydrogen ions in saliva may provide a possible explanation for their caries experience and for the relative effect of this pH variation on the electrolytic composition observed in their saliva. From a biochemical point of view, there are some implications caused by the constant H^+^ raising in saliva, mainly because this behavior could provide a modification of the ionic bioavailability of important ions in the oral cavity such as calcium, phosphate, and fluoride, and consequently affect the exchange of these ions through dental plaque^7,28^. The effect of saliva on *in vivo* plaque pH was investigated by the study of Abelson and Mandel^29^ who found that, in the absence of saliva, the Stephan curve of children with ECC and CF individuals was similar. These authors concluded that stimulated saliva plays a major role in modifying the behavior of plaque pH after a cariogenic challenge and that the effect of stimulated saliva in raising plaque pH after sucrose exposure could quantitatively reflect the dental caries experience. Under this perspective, it is worth mentioning that individuals with ECC express a distinct salivary electrolytic behavior after sucrose mouthwash when compared to caries-free children.

The recognition of the multifactorial etiology of ECC leads to the use of more robust methods to access dental caries susceptibility and the factors underlying the dynamic process of caries initiation. In light of this, our study demonstrated that although the degree of saturation with respect to the DSS HAp and FAp are meaningful measures for previewing teeth susceptibility to dissolution based on the saturation of solutions (under- or supersaturated)^10,30^, in children with ECC these parameters were not shown to be relevant – Table 2. However, the results in Table 3 demonstrate that the salivary calcium is correlated with the degree of saliva saturation, as well as with salivary pH. These outcomes highlight that calcium levels and salivary pH could be possible biomarkers for the chemical phenomena of demineralization and remineralization in children with ECC. Further longitudinal studies should be performed to address this point.

Under the experimental conditions of this study, the salivary pH and buffering capacity were the most important salivary parameters for the occurrence of ECC in a pre-rinse situation. In addition, an increase of 1 μg of Ca^2+^/mL raises by 1.1 times the probability of children to have dental caries. The regression model fitted to explain the post-rinse situation showed that the buffering capacity of saliva is a relevant variable for ECC outcome. On the other hand, after the cariogenic challenge, the pH and the electrolytic composition of saliva did not provide a reliable potential to explain ECC. Theoretically, from a biochemical point of view, all variables included in the models could influence positively or negatively the onset and progression of dental caries^2,31^. However, from a clinical perspective and considering the complexity of the oral environment, the causal effect of sugar exposure must be controlled by potential confounding factors. Thus, these results provide a robust model of how salivary functions behave in a dynamic situation that recognizes the large inter-individual variation.

Several strengths can be found in this research, such as a suitable and specific sample, internal and external validity, and the data collection under customary conditions. However, some limitations should be emphasized. Firstly, the tooth-surface condition and the person-level disease should always be considered as the cause of an eventual biological variance. Secondly, there is no information regarding the children’s dietary behavior, which can be valuable to assess the risk for dental caries development. Thirdly, the experimental design of this study only considered the caries experience (since the early stages of the disease). Thus, it should be considered that the caries activity and the severity of the ECC were not explored in the statistical analysis. Lastly, this study was designed as a cross-sectional study, which makes it difficult to measure causality.

In summary, it was shown that the electrolytic concentration of calcium, phosphate, and fluoride after a 20% sucrose rinse behaves differently when preschool children with ECC and CF individuals were compared. These differences may reflect changes in salivary parameters such as salivary buffering capacity and pH, possibly providing a unique insight into the biochemical dynamic of caries formation in preschoolers.

## Material and Methods

### Ethical approval

All procedures performed in studies involving human participants were in accordance with the ethical standards of the institutional research committee and with the 1964 Helsinki declaration and its later amendments or comparable ethical standards. The Research Ethics Committee of the Piracicaba Dental School/University of Campinas (UNICAMP) approved this study under the protocol CAAE: 70777517.9.0000.5418. Informed consent was obtained from all parents and/or legal guardians of the children included in the study.

### Study design

An experimental, cross-sectional and parallel study was conducted in a sample of 60 children aged 4 to 5 years old. Thus, the study was conducted in two phases:

Phase 1 (*Dental Caries Assessment*): Determination of caries index and division of children into two groups, 01 group of children with ECC (Group ECC, n = 30) and 01 group of caries-free children (Group CF, n = 30).

Phase 2 (*Visible Biofilm Detection and Mouthwash with 20% Sucrose*): After verifying the presence or absence of clinically visible biofilm^32^, saliva samples, before and 5 minutes after 20% sucrose rinse for 1 minute. Saliva collection was performed to determine flow rate, pH, buffering capacity and its electrolytic composition (F^−^, Ca^2+^, and Pi).

### Sample

The sample size was calculated using the application software Gpower 3.1, based on the assumption of a difference between two independent means. The calculation was performed considering an α-value of 0.05, β-value of 0.15, allocation rate of 1/1, and confidence interval of 0.95. The sample size was estimated according to the mean and standard deviation of the salivary concentration of calcium in the saliva of children with ECC and CF children^14^, resulting in 25 for each group. The calculated number (25) was raised to 30 to compensate for possible subject drop-outs during the experiment. Thus, 30 preschoolers per group were included in this study. Children were randomly selected one by one using the Excel lottery method, according to the dental caries experience.

Participants were recruited from daycare centers of the city of Piracicaba, SP, Brazil. The inclusion criteria considered individuals of both sexes, aged 3 to 5 years old, and without systemic diseases. Children whose parents or guardians refused to participate in this study or who did not cooperate with the clinical examinations were excluded. In addition, children with neuromotor or communication difficulties, severe fluorosis, altered salivary flow (below 0.7 mL/min), wearing orthodontic appliance and under antibiotic therapy were also excluded. The volunteers lived in a city with 0.71 mg of F/mL in the drinking water.

### Dental caries assessment

The rater was trained in a population with the same sample age. The training exercise consisted of two stages (theoretical and clinical). The first stage consisted of a theoretical discussion of the caries diagnosis criteria by the World Health Organization^33^, plus the diagnosis of early caries lesions^34^. The second stage was accomplished to assess the consistency of the clinical analysis. The rater and a calibrated pediatric dentist (gold standard) performed the clinical examination in a randomly selected sample of 10 children. Inter-rater reliability was verified using Cohen’s kappa coefficient (κ = 0.89). Diagnosis reproducibility was determined two weeks later by using Cohen’s kappa coefficient (κ = 0.95) calculated from the reevaluation of 60% of the children.

Clinical examinations were performed by the calibrated dentist (the first author). The examinations were carried out in the daycare centers after the properly supervised toothbrushing of each child. During the examination of each child, personal protective equipment as well as sterilized and individual clinical material was used. For diagnosis, the rater used a clinical mirror, a blunt-tipped explorer (Indusbello, Londrina-PR, Brazil), and sterile gauze to remove debris that could prevent caries detection. In addition, to facilitate visualization and correct diagnosis of the early caries lesions (white spot lesions), portable LED flashlight (Sfl5540, Wat Nichia Philips, Brazil) and portable dental equipment with a triple syringe (Odontocase Basic Line, Rio de Janeiro, Brazil) were used.

### Saliva collection

Samples of stimulated saliva were always collected between 9:30 am and 10:30 am at least one and a half hour (1:30) after breakfast. Saliva secretion was mechanically stimulated with a piece of Parafilm® M (Pechiney Plastic Packaging, Inc. Manufactures and Markets Plastic, Chicago, Illinois, USA), and the collection was performed for 5 minutes using graduated tubes. Saliva samples were kept under refrigeration (2 to 8 °C) in an ice container during transportation to the laboratory, where they were centrifuged at 16097.2 g for 15 minutes and then stored at −40 °C until analyses.

### Determination of flow rate, pH and buffering capacity of saliva

Salivary Flow Rate (SFR) was calculated (volume/collection time) and expressed as mL/min. The volume of stimulated saliva was measured in 10 mL graduated cylinders after saliva collection. To determine saliva pH, two hundred microliters of saliva were placed in a tube to determine its initial saliva pH using a pH microelectrode linked to a pH meter pre-calibrated with standard solutions of pH 4 and 7, according to the manufacturer’s instructions (Kasvi, Curitiba, PR, Brazil). Next, Buffering Capacity (BC) was determined in 0.2 mL of saliva and increments of 2 µL of 0.25 M HCl. At each increment, the tube was agitated and the pH determined. The BC was calculated using the following equation: BC = ΔC/ΔpH, where ΔC was the total amount of HCl used to decrease the initial pH to 4.0 and ΔpH is the change in salivary pH. The salivary pH and buffering capacity were determined in the first 30 minutes after collection. During this period, saliva samples were kept in a closed tube to avoid loss of CO_2_.

### Calcium analysis

Calcium concentration in saliva was analyzed by the direct colorimetric method using a microplate spectrophotometer (PowerWave HT, BioTek Instruments, Winooski, VT, USA). Briefly, 25 µL of saliva were pipetted in a 96-well plate to react with a calcium-sensitive reagent (Arsenazo III). The reader was pre-calibrated with a standard curve of calcium carbonate (0–100 µg/mL) and the readings were accomplished using an absorbance of λ 650 nm^35^. The ionic concentration was obtained from the absorbance values with the use of a linear equation (y = ax + b) at a curve fit above 0.98. Samples were analyzed in duplicates and the variation coefficient of the calcium analysis was 0.01.

### Phosphate analysis

Phosphate concentration in saliva was analyzed with the use of the colorimetric method using a microplate spectrophotometer (PowerWave HT, BioTek Instruments, Winooski, VT, USA). In short, 25 µL of saliva were pipetted in a 96-well plate to react with the reducing agents of phosphate (molybdic acid and alpha-aminonaphthol sulfonic acid). The reader was pre-calibrated with a standard curve of phosphate (0–8.27 µg/mL). The readings were accomplished using the absorbance of λ 660 nm^36^. The phosphate ionic concentration was obtained from the absorbance values using a linear equation (y = ax + b) at a curve fit above 0.99. Samples were analyzed in duplicates and the variation coefficient of the analysis was 0.06.

### Fluoride analysis

Fluoride concentration in saliva was measured using the direct method^37^. A fluoride-ion-specific electrode (*BN Model 9409, Orion, Cambridge, MA, USA*) and a potentiometer (*Model 720 Orion, Cambridge, MA, USA*) were used for fluoride analysis, and each sample was analyzed in duplicate (mean reproducibility of readings: 99.75%). Prior to the readings, calibration curves were achieved with fluoride standards from 0.01 to 0.1 mg F/mL and TISAB III (1:10) (Thermo Electron, Fisher Scientific, Walthamam, MA, USA). Fluoride analysis was performed using TISAB III as a buffer sample adjustment. The validity of this method for saliva samples was checked through the addition of known amounts of fluoride to known volumes of saliva in order to test if the increase in F^−^ concentration was equal to the added F^−^ concentration. The results expressed in mV by the potentiometer were converted to fluoride ion concentration using a standard correlation curve (r² > 0.99) based on the linear regression of the calibration curve.

Calcium, phosphate, and fluoride concentrations were expressed as µg/mL.

### Determination of the degree of salivary saturation

The degree of salivary saturation (DSS) with respect to hydroxyapatite (HAp) and fluorapatite (FAp) was calculated based on the solubility product of Hap (10^−58.5^) and Fap (10^−59.6^), the pH and the ionic strength of Ca^2+^, Pi and F^−^ ions. These calculations were based on the equation proposed by Varughese and Moreno^38^:

$$DS={(\frac{IAP}{{K}_{S}})}^{\frac{1}{n}}$$

where IAP is the Ionic Activity Product for HAp [(Ca^2+^)^5^ (PO_4_^3−^)^3^ OH] and FAp [(Ca^2+^)^5^ (PO_4_^3−^)^3^ F], Ks is the solubility product of HAp or FAp, and n = the number of ions in the formula.

This calculation was performed using the application software IONPRODUCT^10^. This software considered a partial pressure of CO_2_ of 0.0004, the atmospheric pressure of 1013.25 bar and 37 °C of temperature. The electroneutrality imbalance was not included in this calculation since the high protein concentration in saliva can promote an electroneutrality imbalance that cannot be properly accounted for the calculation method used in IONPRODUCT.

### Statistical analysis

Data were analyzed using the SPSS package for Windows, version 21.0 (SPSS, Inc., Chicago, IL, USA). The sample distribution was verified using the Shapiro-Wilk test. Comparisons between ECC and CF children were obtained by Student’s t-test and Mann-Whitney test when variables followed Gaussian and non-Gaussian distribution, respectively. Chi-square was used to test whether there was a significant difference between frequencies in one or more categories.

Saliva salivary flow rate, pH, Ca^2+^, Pi and F^−^ concentration, and DSS HAp and DSS FAp did not follow the Gaussian distribution. These variables were transformed using a logarithmic expression with base 10. The one-way repeated measures analysis of variance was used to test the interaction effect between the sucrose rinse (pre- and post-rinse moments) and the independent factor (disease: CF or ECC children) among saliva salivary flow rate, pH, Ca^2+^, Pi and F^−^ concentration, and DSS HAp and DSS FAp. Bonferroni adjustment was applied for multiple comparisons. The Box’ M test was used to prove the equality of multiple variance-covariance matrices considering the 0.001 significance level. The least significant difference (LSD) posthoc test was used to compare means between pre- and post-rinse instants. After the LSD test, Bonferroni adjustment of p-value corrected the level of significance of α.

Pearson’s correlation analysis was used to investigate the intensity of the correlations between physicochemical properties of saliva and the degree of saturation in saliva with respect to hydroxyapatite and fluorapatite. The two-tailed hypothesis was adopted at a 0.05 significance level.

Multiple logistic regression analysis was performed by the forced entry of all independent variables into the equation in a single step. The r² value was adjusted to all independent variables and the alpha significance was set at 0.05. The Odds Ratio and p-value were calculated to perform explanatory models for ECC. Two explanation models were made to verify the relative influence of physiologic salivary parameters and physicochemical properties of saliva (F^−^, Ca^2+^, and Pi) on ECC in children before (Model 1) and after 20% sucrose rinse (Model 2). The DSS with respect to HAp and FAp did not enter as independent variables in the multiple logistic regression to avoid multicollinearity, which could cause a disturbance in this statistical inference.

## Data Availability

All data generated or analyzed during this study are included in this published article (and its Supplementary Information files).

## References

[1] AAPD. Policy on Early Childhood Caries (ECC): Classifications, Consequences, and Preventive Strategies. AAPD Ref Man 2016/2017. 38, 52–54 (2017). 27931420

[2] Pitts NB (2017). Dental caries. Nat. Rev. Dis. Primers..

[3] Takahashi N (2015). Oral Microbiome Metabolism: From “Who Are They?” to “What Are They Doing?”. J. Dent. Res..

[4] Mann and Dickinson, 2006 Mann, A. B. & Dickinson, M. E. Nanomechanics, chemistry and structure at the enamel surface. *Monogr Oral Sci*. **19**, 105–131; 10.1159/0000905882006. 10.1159/000090588 16374031

[5] Featherstone JD (2004). The continuum of dental caries - evidence for a dynamic disease process. J. Dent. Res..

[6] Garcıa-Godoy F, Hicks MJ (2008). Maintaining the integrity of the enamel surface: the role of dental biofilm, saliva and preventive agents in enamel demineralization and remineralization. J. Am. Dent. Assoc..

[7] Dawes C (2008). Salivary flow patterns and the health of hard and soft oral tissues. JADA.

[8] Lenander-Lumikari M, Loimaranta V (2000). Saliva and dental caries. Adv. Dent. Res..

[9] Jain S (2018). Effect of diet modification on salivary parameters and Oratest in high-caries-risk individuals. Int. J. Clin. Pediatr. Dent..

[10] Shellis RP (1988). A microcomputer program to evaluate the saturation of complex solutions with respect to biomineral. Comput. Appl. Biosci..

[11] Singh S (2015). A Saliva as a prediction tool for dental caries: An *in vivo* study. J. Oral. Biol. Craniofacial Res..

[12] Duggal MS, Ciuwla HS, Curzon MEJ (1991). A study of the relationship between trace elements in saliva and dental caries in children. Arch. Oral. Biol..

[13] Preethi BP, Dodawad R, Anand P (2010). Evaluation of flow rate, pH, buffering capacity, calcium, total proteins and total antioxidant capacity levels of saliva in caries-free and caries active children: An *in vivo* study. Ind. J. Clin. Biochem..

[14] Hegde AM, Naik N, Kumari S (2014). Comparison of salivary calcium, phosphate and alkaline phosphatase levels in children with early childhood caries after administration of milk, cheese, and GC tooth mousse: An *in vivo* study. J. Clin. Pediatr. Dent..

[15] Gao X (2016). Salivary biomarkers for dental caries. Periodontol. 2000..

[16] Kubala E (2018). A Review of selected studies that determine the physical and chemical properties of saliva in the field of dental treatment. Biomed. Res. Int..

[17] Margolis, H. C. & Moreno, E. C. Composition of pooled plaque fluid from caries-free and caries positive individuals following sucrose exposure. *J Dent Res*. **71**, 1776–1784; 1177/00220345920710110301 (1992). 10.1177/00220345920710110301 1401439

[18] Tanaka M, Margolis HC (1999). Release of mineral ions in dental plaque following acid production. Arch. Oral. Biol..

[19] Gao XJ (2001). Association of caries activity with the composition of dental plaque fluid. J. Dent. Res..

[20] Astasov-Frauenhoffer M (2017). Exopolysaccharides regulate calcium flow in cariogenic biofilms. Plos One..

[21] Lenton S (2014). A review of the biology of calcium phosphate sequestration with special reference to milk. Dairy. Sci. Technol..

[22] Paes Leme AF (2006). The role of sucrose in cariogenic dental biofilm formation—new insight. J. Dent. Res..

[23] Koo H, Falsetta ML, Klein MI (2013). The exopolysaccharide matrix: a virulence determinant of cariogenic biofilm. J. Dent. Res..

[24] Hwang G (2016). Simultaneous spatiotemporal mapping of *in situ* pH and bacterial activity within an intact 3D microcolony structure. Sci. Rep..

[25] Hicks J, Garcia-Godoy F, Flaitz C (2003). Biological factors in dental caries: role of saliva and dental plaque in the dynamic process of demineralization and remineralization (part 1). J. Clin. Pediatr. Dent..

[26] Larsen MJ, Pearce EIF (2003). Saturation of human saliva with respect to calcium salts. Arch. Oral. Biol..

[27] Animireddy D (2014). Evaluation of pH, buffering capacity, viscosity and flow rate levels of saliva in caries-free, minimal caries and nursing caries children: An *in vivo* study. Contemp. Clin. Dent..

[28] Duckworth RM, Gao XJ (2006). Plaque as a Reservoir for Active Ingredients. Monogr. Oral. Sci..

[29] Abelson DC, Mandel ID (1981). The Effect of Saliva on Plaque pH *in vivo*. Dent. Res..

[30] Larsen JM (1975). Degrees of saturation with respect to apatites in parotid saliva at various pH values. Scand. J. Dent. Res..

[31] Divaris K (2016). Predicting Dental Caries Outcomes in Children: A “Risky” Concept. J. Dent. Res..

[32] Alaluusua S, Malmivirta R (1994). Early plaque accumulation-a sign for caries risk in young children. Community Dent. Oral. Epidemiol..

[33] World Health Organization. Oral health surveys: basic methods - 5th edition. (Geneva, 2013).

[34] Assaf AV (2006). Effect of different diagnostic thresholds on dental caries calibration - a 12-month evaluation. Community Dent. Oral. Epidemiol..

[35] Brown HM (1981). & Rydqvist, Bo. Effect of pH, ionic strength, and Arsenazo III concentration on equilibrium binding evaluated with Ca2+ ion-sensitive electrodes and absorbance measurements. Biophys. J..

[36] Chen PS, Toribara TY, Warner H (1956). Micro determination of phosphorus. Anal. Chem..

[37] Martinez-Mier EA (2011). Development of gold standard ion-selective electrode-based methods for fluoride analysis. Caries Res..

[38] Varughese K, Moreno EC (1981). Crystal growth of calcium apatites in dilute solutions containing fluoride. Calcif. Tiss. Int..

